# Acute Effects of Low-Load Blood Flow Restriction Exercise on Vastus Lateralis Electromyographic Activity in Healthy Young Adults: A Pilot Randomized Study

**DOI:** 10.3390/jfmk11030298

**Published:** 2026-07-28

**Authors:** Rafael Gómez-García, María de los Ángeles Cardero-Durán, Luis Espejo-Antúnez, Carlos Fernández-Morales

**Affiliations:** Department of Medical-Surgical Therapy, Faculty of Medicine and Health Sciences, University of Extremadura, 06006 Badajoz, Spain; rafaelgomgar22@gmail.com (R.G.-G.); luisea@unex.es (L.E.-A.); carlosfm@unex.es (C.F.-M.)

**Keywords:** blood flow restriction, surface electromyography, vastus lateralis, muscle activation, knee extension, physiotherapy, rehabilitation, randomized pilot study

## Abstract

**Background:** Low-load resistance exercise with blood flow restriction (BFR) may increase neuromuscular stimulation while reducing external load. This pilot study examined the acute effects of unilateral knee extension at 20% one-repetition maximum (1RM) with BFR at 80% estimated arterial occlusion pressure (AOP), compared with the same protocol without BFR, on vastus lateralis surface electromyographic (sEMG) activity in healthy young adults. **Methods:** Twenty participants were randomized to receive BFR on either the dominant (*n* = 10) or non-dominant limb (*n* = 10); the contralateral limb completed the non-BFR condition. Both conditions were performed in randomized order during one session, separated by 10 min. Each limb completed 75 repetitions (30-15-15-15) at 20% of its limb-specific 1RM. The prespecified primary outcome was normalized RMS amplitude across 12 protocol windows, analyzed using a linear mixed model. **Results:** Normalized RMS amplitude was higher under BFR (between-condition difference, 21.12%MVIC; 95% CI, 14.54 to 27.71; *p* < 0.001). Exploratory analyses showed higher global mean and peak RMS under BFR, whereas within-protocol RMS change and temporal slope did not differ. The post-exercise percentage reduction in MVIC-derived RMS amplitude was smaller under BFR (−0.6 ± 14.1% vs. −15.4 ± 12.3%; *p* = 0.002). Significant secondary findings remained significant after Holm adjustment. No adverse events were spontaneously reported or observed immediately. **Conclusions:** Under this acute laboratory protocol, individualized BFR was associated with higher vastus lateralis sEMG amplitude than unrestricted exercise. MVIC-derived findings were exploratory and should not be interpreted as evidence of preserved strength or reduced neuromuscular fatigue. Larger studies, including clinical populations, are required.

## 1. Introduction

Preserving or improving lower-limb muscle function is a central goal across a wide range of rehabilitation and conditioning settings [1,2]. Conventional resistance training achieves this through high mechanical loads, but these are frequently inappropriate in post-surgical populations, older adults, or individuals with joint pathology where load tolerance is restricted [1,3]. Low-load resistance exercise combined with blood flow restriction (BFR) has been proposed as a viable alternative in rehabilitation and conditioning settings [1,3]: by applying partial venous occlusion at the proximal thigh via a pneumatic cuff, the method generates a metabolic and neuromuscular environment that low-load exercise alone does not produce [3,4].

The physiological rationale rests on the accumulation of metabolic byproducts—lactate, inorganic phosphate, and hydrogen ions—under conditions of localized hypoxia [5]. This environment may promote the recruitment of higher-threshold motor units to sustain force output and may contribute to the higher surface electromyographic (sEMG) amplitude recorded during BFR compared with matched low-load exercise with free blood flow [6,7]. The relationship between BFR pressure and the resulting neuromuscular response is substantially modulated by the relative cuff pressure expressed as a percentage of individual arterial occlusion pressure (AOP): the discrepancy between the low external load and the elevated internal physiological demand increases with higher relative pressures [8]. Prescribing cuff pressure relative to individual AOP rather than using fixed absolute values is supported by current methodological recommendations [3,9,10].

Quantifying this neuromuscular response via sEMG has been a recurring approach in acute BFR studies. Available evidence confirms that low-load BFR exercise produces higher myoelectric activity than equivalent low-load exercise without restriction [6], while the influence of BFR pressure level on fatigue indices remains inconsistent across studies [11,12,13]. More recently, Davis et al. extended these findings by measuring vastus lateralis RMS amplitude normalized to pre-exercise maximal voluntary isometric contraction (MVIC) in college-aged males performing knee extension protocols at low and moderate loads with BFR and comparing trained and untrained populations [14]. Pairing continuous sEMG recording during exercise with assessment of RMS amplitude during post-exercise MVIC trials allows the exercise-phase excitation response and the immediate post-exercise electromyographic response to be characterized within the same session [14].

Whether this amplitude elevation is sustained across the within-set temporal course and whether BFR attenuates the post-exercise reduction in RMS amplitude during MVIC trials compared to a no-restriction control at a clinically individualized pressure remain poorly characterized in healthy young adults.

The aim of this pilot study was to examine the acute effects of low-load knee extension exercise at 20% 1RM with BFR at 80% of individual AOP, compared to the same protocol without BFR, on vastus lateralis sEMG amplitude assessed with a validated wearable system [15] and on the immediate post-exercise change in RMS amplitude during MVIC trials.

## 2. Materials and Methods

### 2.1. Design

This study was designed as a pilot randomized contralateral within-participant study with two allocation groups and repeated measures. The study was submitted for registration at ClinicalTrials.gov (registration number: NCT07654608; registration date: 1 February 2026). Participants were randomized in a 1:1 ratio to receive BFR on either the dominant or the non-dominant limb. In each participant, the contralateral limb completed the same exercise protocol without BFR. Consequently, each participant contributed measurements under both experimental conditions. The study was reported in accordance with the Consolidated Standards of Reporting Trials (CONSORT) 2025 statement [16] and followed the principles of the Declaration of Helsinki [17]. The study protocol was approved by the Bioethics and Biosafety Committee of the University of Extremadura (approval number: 181/2023; approval date: 15 December 2023). All participants received written and verbal information about the study procedures and provided written informed consent before participation.

### 2.2. Participants

Participants were recruited by convenience sampling through local announcements at the Faculty of Medicine and Health Sciences of the University of Extremadura, Badajoz, Spain. Recruitment took place from 3 March to 15 April 2026. The first participant was enrolled and completed the first experimental session on 3 March 2026, and the final participant was assessed on 15 April 2026. All participants were assessed for eligibility before randomization and provided written informed consent prior to enrollment.

The inclusion criteria were (1) being 18 years of age or older; (2) absence of current pain or active musculoskeletal injury in the lower limbs at the time of assessment; (3) ability to understand the study procedures and follow the exercise protocol; and (4) voluntary agreement to participate in the study. The exclusion criteria were (1) history of neuromuscular disease; (2) cardiovascular disease or uncontrolled hypertension; (3) peripheral vascular disease or previous thromboembolic events; (4) recent lower-limb surgery; (5) skin lesions or dermatological conditions preventing cuff or electrode placement; (6) pregnancy; and (7) any other contraindication to the application of blood flow restriction exercise according to current methodological and safety recommendations [3,9].

### 2.3. Randomization and Blinding

After eligibility assessment and informed consent, participants were randomized in a 1:1 ratio to one of two allocation groups: BFR applied to the dominant limb or BFR applied to the non-dominant limb. The contralateral limb was assigned to the non-BFR condition. The order in which the BFR and non-BFR limb-specific conditions were performed was also randomized for each participant. Participants were enrolled by one investigator after eligibility assessment and provision of informed consent. A second investigator, who was not involved in participant enrollment or experimental assessments, generated the computer-based allocation sequence, including both the limb allocation and the order of the BFR and non-BFR conditions. Limb allocation was generated using a 1:1 ratio and fixed blocks of four. The sequence was stored in a password-protected electronic file that was inaccessible to the enrolling investigator. After each participant had been enrolled, a third researcher accessed the file and communicated the assigned limb and the order in which the two conditions were to be performed. Thus, the upcoming assignment remained concealed from the enrolling investigator until enrollment had been completed.

Due to the nature of the intervention, participants and the researcher supervising the exercise protocol could not be blinded to allocation-group assignment. Surface electromyographic recordings were processed by a researcher blinded to allocation-group assignment. Statistical analyses were performed using coded group labels, and allocation-group assignment was revealed after completion of the analyses.

### 2.4. Assessment

All assessments were performed at the Faculty of Medicine and Health Sciences of the University of Extremadura, Badajoz, Spain, using a standardized laboratory protocol. Each participant completed the low-load knee extension protocol with both lower limbs according to the randomized limb-specific allocation: one limb under BFR and the contralateral limb without BFR. Both limb-specific conditions were completed during the same experimental session in randomized order, with a 10 min rest period between conditions, consistent with a previously published randomized contralateral protocol [18]. The BFR cuff was fully deflated and removed immediately after completion of the BFR condition. Baseline sociodemographic and anthropometric data, including age, height, and body mass, were recorded before the experimental procedure.

For each assessed limb, sEMG activity during pre-exercise maximal voluntary isometric contraction (MVIC) of the knee extensors was recorded before the exercise protocol. The corresponding limb then completed the low-load knee extension protocol under its allocated condition while vastus lateralis sEMG activity was recorded continuously. Immediately after completion of the exercise protocol, sEMG activity was recorded during the post-exercise MVIC assessment.

All assessments were conducted under the same environmental conditions and following the same procedures for both experimental conditions.

#### Surface Electromyography Recording and Outcome Measures

Surface electromyographic (sEMG) activity was recorded from the vastus lateralis of each assessed limb using the mDurance^®^ surface electromyography system (mDurance Solutions SL, Granada, Spain). The system uses a portable Shimmer3 EMG unit with two sEMG channels, a sampling rate of 1024 Hz, 24-bit signal resolution, and an overall amplification of 100–10,000 *V*/*V*. The mDurance^®^ mobile application receives data from the sensor and sends them to a cloud service, where sEMG signals are stored, filtered, and analyzed [15]. Raw sEMG signals were processed according to the mDurance^®^ methodology, using a fourth-order Butterworth band-pass filter with cut-off frequencies of 20–450 Hz and root mean square (RMS) smoothing with a 0.025 s window and 0.0125 s overlap. The mDurance^®^ system has shown validity for measuring muscle activity during dynamic contractions when compared with a reference surface electromyography system [15]. Before electrode placement, the skin was cleaned to reduce impedance and improve signal quality. The electrode was placed on the vastus lateralis according to SENIAM recommendations, at two-thirds of the line between the anterior superior iliac spine and the lateral border of the patella [19].

Before the exercise protocol, participants performed pre-exercise maximal voluntary isometric contraction (MVIC) trials to obtain the reference RMS amplitude for normalization of the electromyographic signal. Pre- and post-exercise MVIC assessments were performed separately for each assessed lower limb. Each assessment consisted of three maximal isometric knee extension trials separated by 120 s of rest, and participants were verbally instructed to perform each trial with maximal effort. The highest RMS value obtained across the three pre-exercise trials was used as the reference for EMG normalization, whereas the highest RMS value obtained across the three post-exercise trials was used for the post-exercise comparison. RMS amplitude recorded during the exercise protocol was normalized to the pre-exercise MVIC-derived RMS amplitude obtained from the corresponding limb. The post-exercise assessment began immediately after completion of the protocol. Normalization to a reference MVIC was used to facilitate between-participant and between-condition comparisons of electromyographic amplitude [20].

The single primary outcome was normalized RMS amplitude of the vastus lateralis during the knee extension protocol, expressed as a percentage of pre-exercise MVIC (%MVIC). RMS values were extracted from predefined analysis windows within each set: each window comprised five consecutive complete repetitions. In Set 1, the initial, middle, and final windows corresponded to repetitions 1–5, 13–17, and 26–30, respectively. In Sets 2, 3, and 4, these windows corresponded to repetitions 1–5, 6–10, and 11–15, respectively. RMS amplitude was calculated across the complete repetition cycles, without separating the concentric and eccentric phases. For each participant, summary sEMG variables were calculated separately for the BFR and non-BFR conditions: global mean RMS (%MVIC), peak RMS (%MVIC), within-protocol RMS change (%MVIC), and temporal RMS slope (%MVIC/point). Global mean RMS was calculated separately for each participant and condition as the arithmetic mean of the 12 normalized RMS values obtained across the protocol, comprising three analyzed windows in each of the four sets. For descriptive set-level values, the initial, middle, and final normalized RMS windows were averaged within each set for each participant and condition, and these participant-level set means were subsequently averaged across participants. Peak RMS was defined as the highest normalized RMS value recorded across the 12 protocol windows for each participant and condition. Within-protocol RMS change was calculated as the difference between the final analyzed window of Set 4 and the initial analyzed window of Set 1. Temporal RMS slope was calculated as the linear change in normalized RMS across the analyzed protocol points.

Complementary outcomes derived from the maximal voluntary isometric contraction trials included pre-exercise RMS amplitude during MVIC, post-exercise RMS amplitude during MVIC, absolute change in RMS amplitude during MVIC (post-exercise minus pre-exercise, µV), and percentage change relative to the pre-exercise MVIC RMS amplitude.

### 2.5. Intervention

Each participant completed the low-load knee extension protocol with both lower limbs according to the randomized limb-specific allocation. The protocol consisted of 75 repetitions distributed across four sets, following a 30-15-15-15 repetition scheme, with 30 s of rest between sets [3,10]. Before the experimental protocol, unilateral one-repetition maximum (1RM) for knee extension was determined separately for each lower limb using a standardized direct 1RM testing procedure, which has shown good-to-excellent test–retest reliability [21]. The external load for each limb was then set at 20% of its corresponding limb-specific 1RM. This protocol was selected because low-load BFR resistance exercise is commonly prescribed using loads of 20–40% 1RM, fixed repetition schemes of 75 repetitions, and short inter-set rest intervals [3]. Similar fixed-repetition knee extension protocols have been used in previous studies examining electromyographic activity and acute fatigue responses during low-load exercise with BFR [6,11,12], as well as in acute neuromuscular protocols combining low-load knee extension with BFR at 80% AOP [22].

Participants performed the exercise in a seated position, with repeated knee extensions of the assessed limb. They were instructed to complete the full number of repetitions in each set while maintaining a controlled execution rhythm and avoiding compensatory trunk or hip movements. Repetition cadence was standardized using an audible metronome, with 1 s assigned to the concentric phase and 1 s to the eccentric phase. Instructions, verbal encouragement, supervision, and investigator interaction were standardized across both conditions, and no condition-specific motivational cues were provided. Movement artifacts were minimized through skin preparation, standardized electrode placement, seated positioning, direct supervision, and verbal correction of compensatory trunk or hip movements. Signal filtering was applied as described above. The same exercise protocol, load, number of repetitions, and rest intervals were used in both conditions.

In the BFR condition, a pneumatic blood flow restriction cuff (Akrafit BFR, Antalya, Turkey, reference AF2022; large cuff dimensions: 82 × 10.5 cm) connected to a manual pump was placed on the proximal portion of the thigh of the exercised limb.

Before AOP assessment, participants rested for 10 min in the seated position used for the exercise protocol, with the assessed limb relaxed. Estimated AOP was determined twice using the same cuff subsequently used during exercise, with a 5 min interval between measurements. The cuff was progressively inflated at approximately 10 mmHg every 10 s [23]. The popliteal pulse, detected over the popliteal artery in the popliteal fossa, was monitored by manual auscultation. The pressure at which the pulse was no longer audible was recorded as the estimated AOP. When the two measurements differed by no more than 20 mmHg, their mean was used. When the difference exceeded 20 mmHg, a third measurement was performed, and the mean of the three measurements was calculated. All AOP measurements were performed by the same assessor using the same cuff and auscultatory equipment.

During the BFR condition, the cuff was inflated to 80% of the estimated individual AOP using the manual pump. The pump and pressure gauge remained connected throughout the four exercise sets and the inter-set rest periods. Cuff pressure was monitored continuously on the pressure gauge, and any deviation from the prescribed pressure was corrected manually. The cuff remained inflated during the 30 s inter-set rest periods and was completely deflated and removed immediately after the final set. This individualized approach was selected because current methodological recommendations support prescribing BFR pressure relative to each participant’s AOP rather than using a fixed absolute pressure [3,10]. The use of 80% AOP was selected because it corresponds to the upper limit of the recommended individualized pressure range for lower-limb BFR resistance exercise [3] and has been used in previous acute low-load BFR studies assessing neuromuscular and electromyographic responses [6].

In the non-BFR condition, participants performed the same low-load knee extension protocol without blood flow restriction. No cuff pressure was applied during the exercise protocol. Surface electromyographic activity of the vastus lateralis was recorded continuously throughout the four exercise sets in both conditions.

Adverse events were monitored through continuous investigator observation and spontaneous participant reporting during the experimental procedures and the immediate post-exercise period. Any reported or observed event was recorded.

### 2.6. Sample Size

The sample size was estimated a priori using G*Power 3.1 [24]. Because the planned primary analysis was based on repeated measurements of normalized RMS amplitude, an F-test family procedure for repeated-measures ANOVA with a between-participant allocation factor was used as a conventional approximation to the planned longitudinal analysis. The calculation was based on two allocation groups, 12 repeated measurements, a large expected effect size (f = 0.50), an alpha level of 0.05, 80% statistical power, and an assumed correlation among repeated measures of 0.50. These assumptions were informed by the comparable repeated-measures study by Fatela et al. [11], which reported marked differences in RMS amplitude during low-load knee extension at different relative BFR pressures. The effect size was selected to represent the large response observed in that study, whereas the correlation of 0.50 was adopted as a moderate planning assumption because the correlation required for the present 12 repeated measurements was not directly reported. This yielded a target sample size of 20 participants. The calculation was intended to guide recruitment and should not be interpreted as establishing definitive confirmatory power for the final linear mixed model. The final sample comprised 20 participants, with 10 participants in each randomized allocation group. All participants contributed measurements under both BFR and non-BFR conditions.

### 2.7. Statistical Analysis

Statistical analyses were performed using IBM SPSS Statistics for Windows, version 23.0 (IBM Corp., Armonk, NY, USA). Descriptive data are presented as mean ± standard deviation. Participant characteristics were summarized descriptively using one observation per participant.

The single primary outcome was normalized RMS amplitude of the vastus lateralis, expressed as a percentage of pre-exercise MVIC (%MVIC). To analyze the longitudinal behavior of normalized RMS amplitude during the exercise protocol, a linear mixed model was estimated using restricted maximum likelihood. Condition (BFR vs. non-BFR), set (Set 1 to Set 4), within-set moment (initial, middle, and final), and all two- and three-way interactions among these factors were entered as fixed effects. Condition was modeled as a within-participant factor because each participant contributed one limb under each condition. The allocation group—BFR applied to the dominant or non-dominant limb—was included as a between-participant fixed factor, and the condition × allocation group interaction was examined. Random intercepts were specified for participant and for limb nested within participant. No random slopes were included. A scaled-identity residual covariance structure was used. Model-based standard errors were calculated, fixed effects were evaluated using Type III F tests, and denominator degrees of freedom were estimated using the Satterthwaite approximation. As a sensitivity analysis, normalized RMS amplitude was averaged across the 12 protocol windows for each participant and condition, and the resulting participant-level means were compared using a paired-samples *t*-test.

Summary sEMG outcomes were calculated separately for each participant and condition, including global mean RMS (%MVIC), peak RMS (%MVIC), within-protocol RMS change (%MVIC), and temporal RMS slope (%MVIC/point). These participant-level outcomes were compared between conditions using paired-sample *t*-tests, with each participant contributing one value under each condition. No covariates, weighting procedures, or repeated window-level observations were included in these analyses.

RMS amplitude during MVIC trials was analyzed using a linear mixed model including condition, time (pre-exercise vs. post-exercise), and the condition × time interaction as fixed effects, with participant included as a random effect. Absolute and percentage changes were calculated separately for each condition and compared using paired-sample *t*-tests. Effect estimates are reported as between-condition mean differences with 95% confidence intervals. Mean differences were calculated as BFR minus non-BFR. Model assumptions were assessed using residual quantile–quantile plots, residuals-versus-fitted plots, and influence diagnostics. No relevant violations were identified. The longitudinal mixed-model analysis of normalized RMS amplitude was the prespecified primary analysis. All remaining outcome analyses were considered secondary and exploratory. As a sensitivity analysis for multiplicity, Holm-adjusted *p*-values were calculated across the nine secondary inferential tests.

Statistical significance was set at *p* < 0.05.

## 3. Results

### 3.1. Participants and Baseline Characteristics

**Figure 1:**
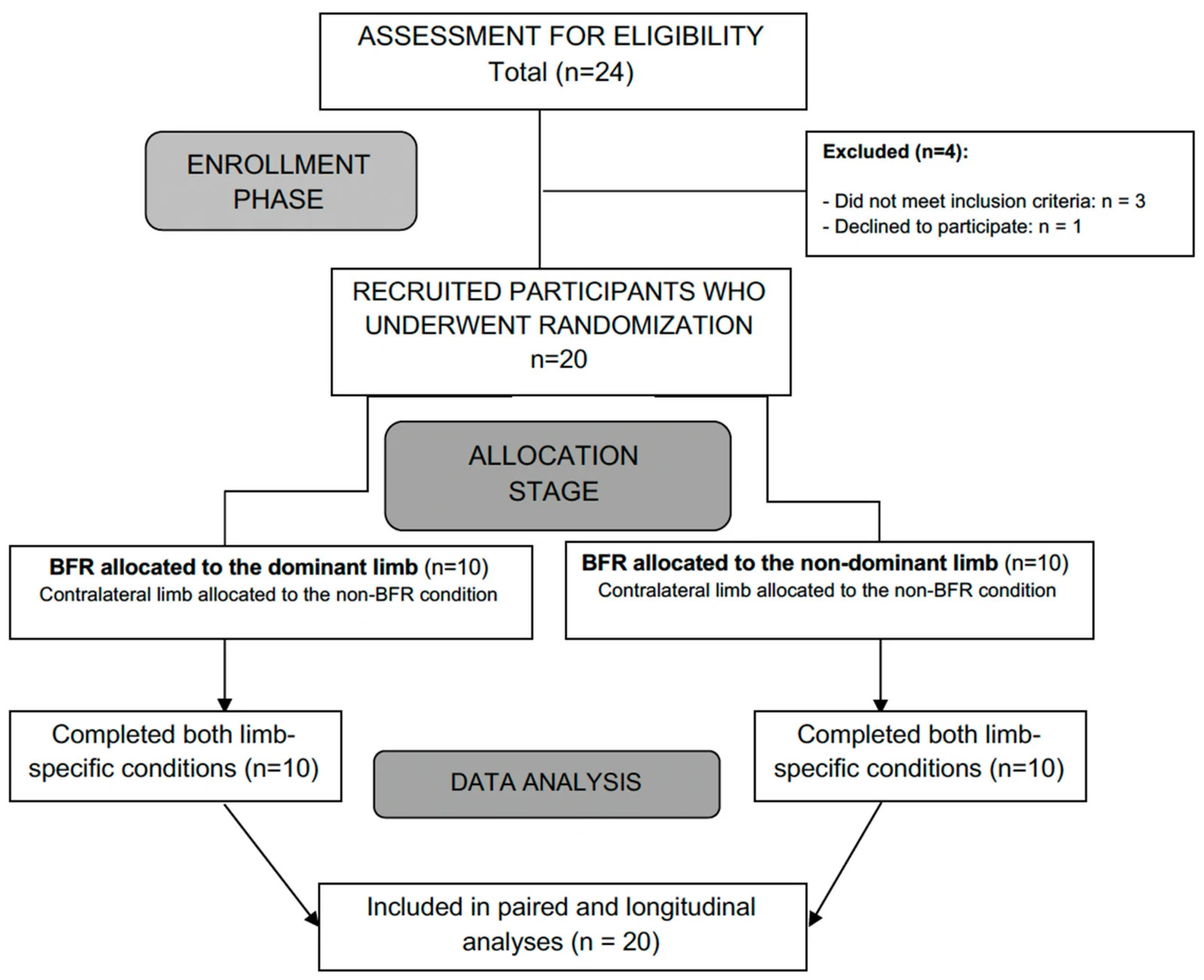
Participant flow and randomized limb allocation. BFR: blood flow restriction.

Twenty healthy young adults were included and randomized to one of two allocation groups. Ten participants were allocated to receive BFR on the dominant limb, whereas the remaining ten received BFR on the non-dominant limb. In both allocation groups, the contralateral limb completed the same exercise protocol without BFR. Therefore, all participants contributed measurements under both BFR and non-BFR conditions. The overall sample comprised 13 men and 7 women, with a mean age of 22.4 ± 3.0 years, a mean height of 1.73 ± 0.07 m, and a mean body mass of 72.0 ± 14.0 kg. In the BFR condition, the estimated individual AOP was 177.9 ± 21.8 mmHg, with a range of 145–214 mmHg. The corresponding cuff pressure applied during exercise was estimated at 142.3 ± 17.4 mmHg, ranging from 116 to 171 mmHg. The participant flow is shown in Figure 1.

No adverse events were spontaneously reported by participants or observed by the supervising investigator during or immediately after the experimental procedures.

### 3.2. Normalized sEMG Amplitude During the Knee Extension Protocol

**Figure 2:**
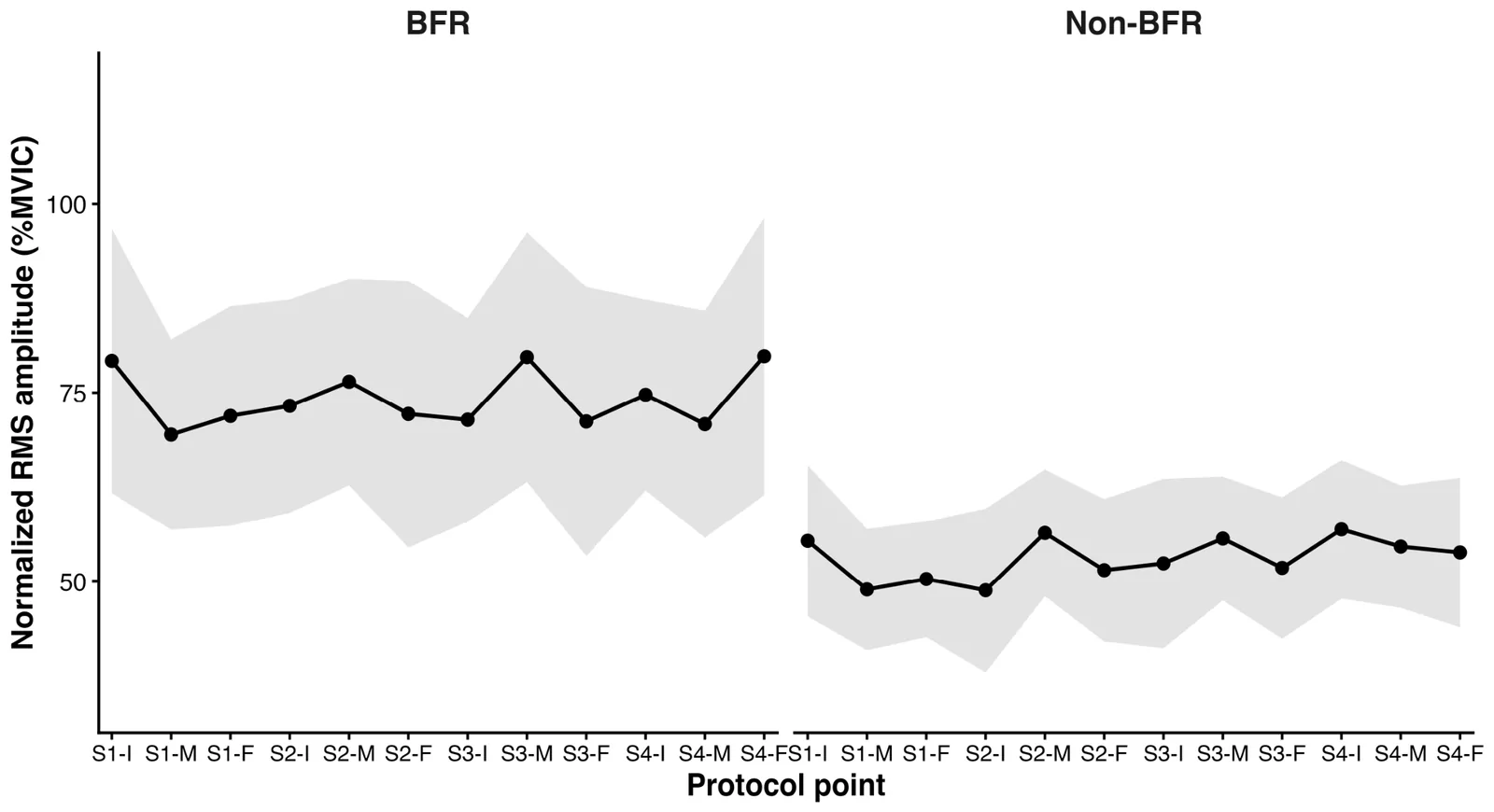
Normalized vastus lateralis RMS amplitude during the low-load knee extension protocol. Values represent mean RMS amplitude normalized to the pre-exercise MVIC and are shown across the initial, middle, and final analyzed windows of each set. Shaded areas represent 95% confidence intervals. BFR: blood flow restriction; MVIC: maximal voluntary isometric contraction; RMS: root mean square.

The primary longitudinal mixed-model analysis showed a significant main effect of condition on normalized vastus lateralis RMS amplitude, with higher values in the BFR condition than in the non-BFR condition (between-condition difference, 21.12%MVIC; SE, 3.13; 95% CI, 14.54 to 27.71; F(1, 18) = 45.41; *p* < 0.001). Neither the main effect of the allocation group nor the condition × allocation group interaction was statistically significant (*p* = 0.984 and *p* = 0.965, respectively). No significant main effects of set (*p* = 0.512) or within-set moment (*p* = 0.674) were observed. A significant set × within-set moment interaction was identified (*p* = 0.010), whereas the condition × set (*p* = 0.943), condition × within-set moment (*p* = 0.855), and condition × set × within-set moment (*p* = 0.651) interactions were not statistically significant. The estimated variance components were 498.54 (%MVIC)^2^ for participant, 82.23 (%MVIC)^2^ for limb nested within participant, and 192.15 (%MVIC)^2^ for the residual. A participant-level sensitivity analysis based on the mean RMS amplitude across the 12 protocol windows yielded a similar between-condition difference (21.12%MVIC; 95% CI, 14.73 to 27.51; t(19) = 6.92; *p* < 0.001). The mean RMS pattern across conditions, sets, and within-set points is displayed in Figure 2.

Mean RMS amplitude across Sets 1–4 was 73.6, 74.0, 74.1, and 75.1%MVIC, respectively, in the BFR condition, and 51.6, 52.3, 53.3, and 55.1%MVIC, respectively, in the non-BFR condition. In exploratory secondary analyses, global mean RMS was higher in the BFR condition than in the non-BFR condition (74.2 ± 29.3%MVIC vs. 53.1 ± 16.5%MVIC; mean difference, 21.12%MVIC; 95% CI, 14.73 to 27.51; *p* < 0.001). Peak RMS was also higher under BFR (87.6 ± 42.6%MVIC vs. 63.8 ± 25.1%MVIC; mean difference, 23.77%MVIC; 95% CI, 14.37 to 33.17; *p* < 0.001). Neither within-protocol RMS change nor temporal RMS slope differed significantly between conditions (Table 1). Complete fixed-effect tests and covariance-parameter estimates are provided in Table S1.

### 3.3. Post-Exercise Change in RMS Amplitude During MVIC Trials

In exploratory analyses of MVIC-derived outcomes, a significant condition × time interaction was observed for RMS amplitude during MVIC trials (F(1, 19) = 10.01; *p* = 0.005). Pre-exercise RMS amplitude did not differ significantly between conditions (259.0 ± 115.5 µV under BFR vs. 242.7 ± 50.3 µV under non-BFR; mean difference, 16.30 µV; 95% CI, −45.35 to 77.96; *p* = 0.586). Post-exercise RMS amplitude was higher under BFR, although the between-condition difference was not statistically significant (250.0 ± 95.8 µV vs. 202.2 ± 33.7 µV; mean difference, 47.80 µV; 95% CI, −5.54 to 101.14; *p* = 0.076).

**Figure 3:**
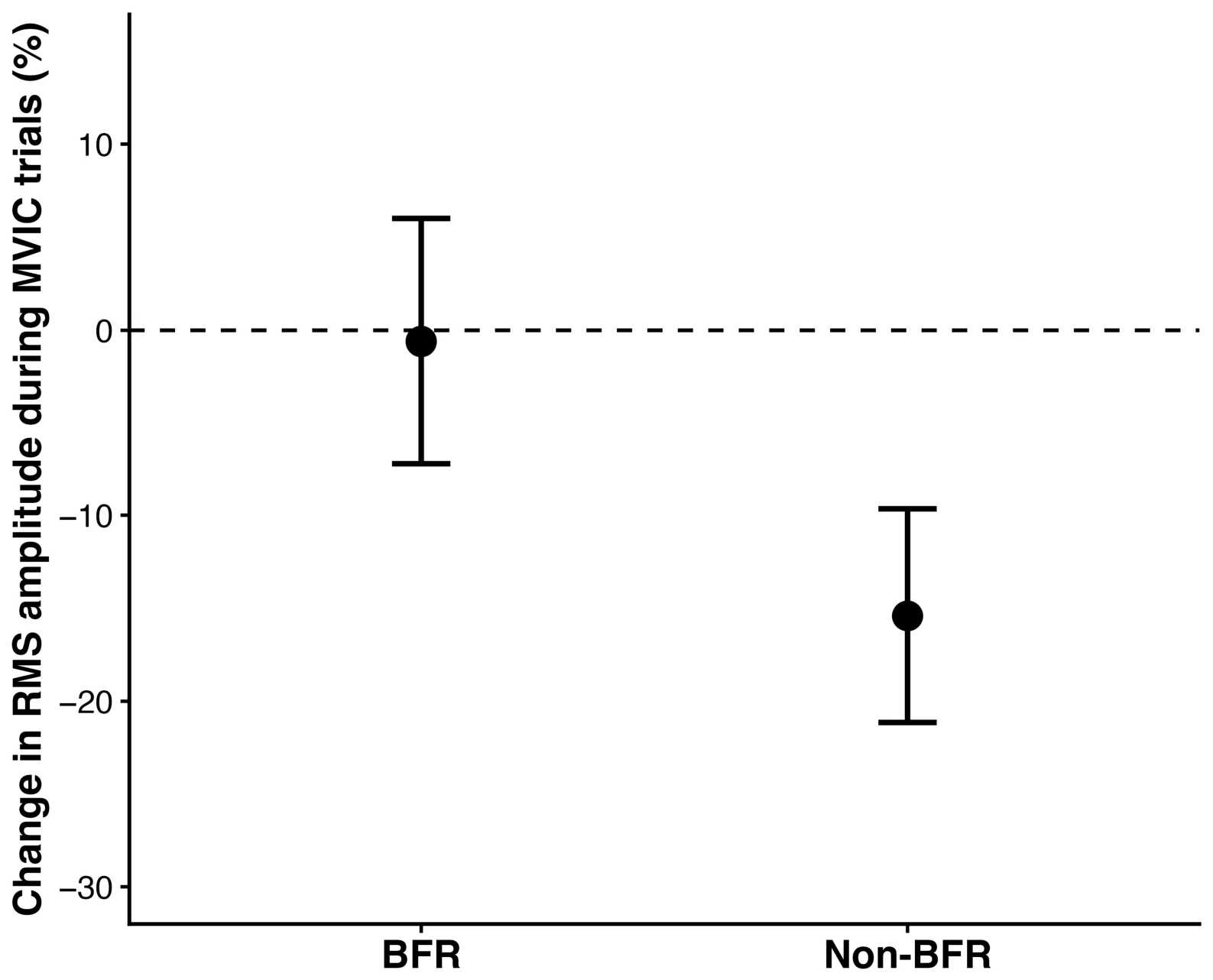
Percentage change in RMS amplitude during maximal voluntary isometric contraction trials after the knee extension protocol. Points represent mean values, and error bars represent 95% confidence intervals. BFR: blood flow restriction; MVIC: maximal voluntary isometric contraction; RMS: root mean square.

The absolute change in RMS amplitude during MVIC trials was −8.9 ± 33.7 µV under BFR and −40.4 ± 33.4 µV under non-BFR (mean difference, 31.50 µV; 95% CI, 10.67 to 52.34; *p* = 0.005). Percentage change was also significantly different between conditions (−0.6 ± 14.1% vs. −15.4 ± 12.3%; mean difference, 14.80 percentage points; 95% CI, 6.33 to 23.27; *p* = 0.002), as shown in Figure 3. The complete MVIC-derived outcomes are summarized in Table 2.

In the Holm-adjusted multiplicity sensitivity analysis, global mean RMS, peak RMS, the condition × time interaction, absolute change in MVIC-derived RMS amplitude, and percentage change remained statistically significant (adjusted *p* < 0.001, < 0.001, 0.031, 0.031, and 0.012, respectively). All other secondary comparisons remained non-significant.

## 4. Discussion

The primary longitudinal analysis showed higher normalized vastus lateralis RMS amplitude during low-load knee extension with BFR at 80% individual AOP than during the same protocol without restriction. Exploratory secondary analyses showed higher global mean and peak RMS and a smaller post-exercise reduction in MVIC-derived RMS amplitude under BFR, whereas within-protocol RMS change and temporal RMS slope did not differ between conditions. The statistically significant secondary findings remained significant after Holm adjustment but should be considered exploratory and hypothesis-generating.

Higher sEMG amplitude during low-load BFR knee extension has been reported across a range of protocols and restriction pressures. At 20% 1RM, pressure-dependent increases in muscle activation and neuromuscular fatigue have been documented across restriction levels from 40% to 80% AOP, with the highest relative pressures producing the largest electromyographic and metabolic responses [11]. Greater quadriceps activation during low-intensity contractions with restricted blood flow compared to equivalent unrestricted contractions has been observed in both healthy and cardiovascular populations [7,25], a finding corroborated at the meta-analytic level [6].

Beyond amplitude, BFR at 20% 1RM knee extension has been shown to recruit motor units with higher action potential amplitude and increase their firing rates, a pattern attributed to the accelerated fatigue of lower-threshold units under localized hypoxia and the consequent drive to engage higher-threshold fibers [26]. The present RMS data are compatible with this interpretation but do not demonstrate it. Surface EMG amplitude may be influenced by multiple physiological and methodological factors and cannot be interpreted as a direct measure of motor-unit recruitment [20]. Because tissue oxygenation and metabolic accumulation were not directly assessed, the higher RMS amplitude cannot be attributed specifically to greater recruitment of higher-threshold motor units.

The magnitude of the RMS response is relevant in relation to the restriction pressure applied. Higher relative cuff pressures produce greater reductions in tissue oxygenation and larger submaximal EMG responses during low-load knee extension [12], and increasing restriction pressure has been associated with augmented muscle activation, though the response interacts with external load and exercise volume [13]. At 80% AOP, the applied restriction may have contributed to the consistent amplitude elevation observed across all four sets without a divergent temporal pattern between conditions.

In fixed-repetition BFR protocols using the 30-15-15-15 scheme, sEMG amplitude has been reported to plateau after the first two to three sets rather than increase progressively [27]. In the present study, no condition-related interaction was statistically significant, indicating that BFR did not produce a distinct progressive temporal pattern. However, the significant set × within-set moment interaction suggests that the pooled within-protocol pattern was not entirely uniform. The physiological basis of this variation cannot be determined from the present measurements.

The post-exercise change in RMS amplitude during MVIC trials warrants separate interpretation. The absence of a greater reduction in MVIC-derived RMS amplitude in the BFR condition does not contradict the higher exercise-phase RMS amplitude: protocols taken to concentric torque failure produce significant MVC decrements under both restricted and unrestricted conditions [28], whereas fixed-volume protocols that do not reach failure show attenuated fatigue regardless of restriction [29]. However, these studies included mechanical or performance-based outcomes that were not assessed in the present study. The percentage change in MVIC-derived RMS amplitude observed here was significantly smaller after BFR than after non-BFR exercise, but this electromyographic difference cannot be interpreted as evidence of preserved maximal strength or reduced neuromuscular fatigue because force or torque was not recorded.

### 4.1. Clinical Implications

In this single-session laboratory study, individualized BFR increased vastus lateralis sEMG amplitude during low-load knee extension without increasing external load. This finding primarily demonstrates an acute difference in electrical muscle activity under the specific protocol tested and should not be interpreted as evidence of clinical effectiveness. Because the study included healthy young adults and did not assess strength, pain, functional performance, adherence, or longer-term adaptations, its relevance to rehabilitation remains indirect. These findings provide a rationale for future studies in clinical populations for whom high-load resistance exercise may be inappropriate or poorly tolerated [1,30], including after anterior cruciate ligament reconstruction [31].

### 4.2. Limitations

The sample was small, limiting the precision of both the effect estimates and the variance-component estimates from the mixed model, and the results should be read as those of a pilot study. The approximate sample-size calculation was intended to guide recruitment and does not establish definitive confirmatory power. Participants were healthy young adults recruited by convenience sampling, limiting extrapolation to clinical or older populations. The design assessed a single acute session, so no conclusions can be drawn regarding training adaptations. Although the order of the limb-specific conditions was randomized and a 10 min rest interval was provided, residual systemic or contralateral effects associated with the acute BFR stimulus cannot be completely excluded. The absence of a sham-BFR condition, together with the inability to blind participants and supervising investigators, prevents sensory, discomfort, expectancy, perceived-exertion, or movement-strategy effects associated with cuff application from being separated from the physiological effects of vascular restriction. Consequently, the observed between-condition differences cannot be attributed exclusively to vascular restriction. Additional limitations include the use of manual auscultation rather than Doppler ultrasound to estimate AOP and the assessment of sEMG activity only from the vastus lateralis. Some outcomes showed substantial interindividual variability, which should be considered when interpreting the estimates. Outcomes were restricted to sEMG amplitude and MVIC-derived RMS. These measures characterize muscle electrical activity but do not directly quantify force production, neuromuscular performance, or clinically meaningful adaptations. Direct measures of strength or torque, muscle oxygenation, blood lactate concentration, or perceived exertion were not obtained. Consequently, maximal mechanical effort could not be independently verified during either the pre- or post-exercise MVIC assessments. Accordingly, the physiological mechanisms underlying the observed RMS responses cannot be established from the present data, and the post-exercise change in MVIC-derived RMS amplitude cannot be interpreted as a direct measure of maximal strength or neuromuscular fatigue. Although the principal secondary findings remained statistically significant after Holm adjustment, these analyses were exploratory and should be considered hypothesis-generating. Future studies should combine sEMG with mechanical and physiological outcomes, since objective physiological measures have been used to characterize treatment-related responses in other physiotherapy interventions [32].

## 5. Conclusions

In this pilot randomized contralateral study, low-load knee extension at 20% 1RM with BFR at 80% individual AOP was associated with higher vastus lateralis sEMG amplitude than the same protocol without restriction. Exploratory secondary analyses identified a smaller post-exercise reduction in MVIC-derived RMS amplitude under BFR, although this electromyographic finding cannot be interpreted as evidence of preserved maximal strength or reduced neuromuscular fatigue. These findings are limited to an acute laboratory protocol and do not establish clinical effectiveness. Confirmation in larger studies and clinical populations is required.

## Figures and Tables

**Table 1:** Summary of sEMG outcomes during the knee extension protocol.

Variable	BFR	Non-BFR	Mean Difference (95% CI)	*p*-Value
Global mean RMS (%MVIC)	74.2 ± 29.3	53.1 ± 16.5	21.12 (14.73 to 27.51)	<0.001
Peak RMS (%MVIC)	87.6 ± 42.6	63.8 ± 25.1	23.77 (14.37 to 33.17)	<0.001
Within-protocol RMS change (%MVIC)	0.60 ± 6.49	−1.54 ± 3.87	2.15 (−1.04 to 5.34)	0.175
Temporal RMS slope (%MVIC/point)	0.13 ± 1.03	0.33 ± 0.68	−0.20 (−0.87 to 0.47)	0.543

Values are presented as mean ± standard deviation across participants. All 20 participants contributed one value under each condition, and between-condition comparisons were paired. Mean differences were calculated as BFR minus non-BFR. CI: confidence interval. BFR: blood flow restriction; RMS: root mean square; MVIC: maximal voluntary isometric contraction; sEMG: surface electromyography.

**Table 2:** Pre- and post-exercise RMS amplitude during maximal voluntary isometric contraction trials by condition.

Variable	BFR	Non-BFR	Mean Difference (95% CI)	*p*-Value
Pre-exercise RMS amplitude during MVIC trials (µV)	259.0 ± 115.5	242.7 ± 50.3	16.30 (−45.35 to 77.96)	0.586
Post-exercise RMS amplitude during MVIC trials (µV)	250.0 ± 95.8	202.2 ± 33.7	47.80 (−5.54 to 101.14)	0.076
Absolute change in RMS amplitude during MVIC trials (µV)	−8.9 ± 33.7	−40.4 ± 33.4	31.50 (10.67 to 52.34)	0.005
Percentage change in RMS amplitude during MVIC trials (%)	−0.6 ± 14.1	−15.4 ± 12.3	14.80 (6.33 to 23.27)	0.002

Values are presented as mean ± standard deviation across participants. All 20 participants contributed measurements under both conditions, and between-condition comparisons were paired. Mean differences were calculated as BFR minus non-BFR. CI: confidence interval. BFR: blood flow restriction; MVIC: maximal voluntary isometric contraction; RMS: root mean square.

## Data Availability

The data supporting the findings of this study are available from the corresponding author upon reasonable request. The data are not publicly available due to privacy and ethical restrictions related to human participant data.

## Supplementary Materials

The following supporting information can be downloaded at https://www.mdpi.com/article/10.3390/jfmk11030298/s1: Table S1. Type III tests of fixed effects from the longitudinal linear mixed model.
