# Supplementary material for: Acute Effects of Low-Load Blood Flow Restriction Exercise on Vastus Lateralis Electromyographic Activity in Healthy Young Adults: A Pilot Randomized Study
Source: J Funct Morphol Kinesiol. 2026 Jul 28;11(3):298. doi: 10.3390/jfmk11030298 (PMC13511683; doi:10.3390/jfmk11030298)
Supplement: Supplementary file 1 [file jfmk-11-00298-s001.zip › jfmk-4413760-supplementary.pdf]

Table S1. Type III tests of fixed effects from the longitudinal linear mixed model.

Fixed effect	Numerator df	Denominator df	F	p-value
Condition	1	18	45.41	<0.001
Allocation group	1	18	<0.001	0.984
Condition × allocation group	1	18	0.002	0.965
Set	3	418	0.77	0.512
Within-set moment	2	418	0.39	0.674
Condition × set	3	418	0.13	0.943
Condition × within-set moment	2	418	0.16	0.855
Set × within-set moment	6	418	2.84	0.010
Condition × set × within-set moment	6	418	0.70	0.651

The model was estimated using restricted maximum likelihood. Denominator degrees of freedom were calculated using the Satterthwaite approximation. Random intercepts were specified for participant and limb nested within participant. Variance estimates were 498.54 $(\%MVIC)^2$ for participant, 82.23 $(\%MVIC)^2$ for limb nested within participant, and 192.15 $(\%MVIC)^2$ for the residual. BFR: blood flow restriction; MVIC: maximal voluntary isometric contraction.
